# *Caloboletus luolongensis* sp. nov. and supplementary notes on *Chenrenyua longispora* (*Boletaceae*) from Guizhou Province, China

**DOI:** 10.3897/mycokeys.142.196913

**Published:** 2026-10-26

**Authors:** Xing Mo, Zhuo-Qiong Qi, Zhi-Qi Zhang, Rong Dai, Ya-Dong Li, Yun Pei, Jing-Wei Li, Ting-Chi Wen, Wan-Ping Zhang, Nian-Kai Zeng, Xiu-Hong Xu

**Affiliations:** 1 College of Agriculture, Guizhou University, Guiyang 550025, China College of Agriculture, Guizhou University Guiyang China; 2 Institute of Vegetable Industry Technology Research, Guizhou University, Guiyang 550025, China Institute of Vegetable Industry Technology Research, Guizhou University Guiyang China; 3 Guizhou Modern Seed Industry Group Co., Ltd., Guiyang 550000, China Guizhou Modern Seed Industry Group Co., Ltd. Guiyang China; 4 The Engineering Research Center of Southwest Bio-Pharmaceutical Resources, Ministry of Education, Guizhou University, Guiyang 550025, China The Engineering Research Center of Southwest Bio-Pharmaceutical Resources, Ministry of Education, Guizhou University Guiyang China; 5 Ministry of Education Key Laboratory for Ecology of Tropical Islands, Key Laboratory of Tropical Animal and Plant Ecology of Hainan Province, College of Life Sciences, Hainan Normal University, Haikou 571158, China Ministry of Education Key Laboratory for Ecology of Tropical Islands, Key Laboratory of Tropical Animal and Plant Ecology of Hainan Province, College of Life Sciences, Hainan Normal University Haikou China

**Keywords:** *
Boletales
*, morphology, multigene phylogeny, new species, taxonomy

## Abstract

*Caloboletus luolongensis***sp. nov**. is described from Guizhou Province, southwestern China, based on morphological characters and multilocus phylogenetic analyses. It is characterized by a rugose pileus with a slightly incurved margin, a longitudinally reticulate stipe that is rose-red over its middle and lower parts, and a context that bruises blue when injured. Supplementary notes on the known bolete *Chenrenyua longispora* are also provided based on newly collected materials from Guizhou. Phylogenetic reconstructions were performed on a combined dataset of nrLSU, nrITS, TEF1-α, and RPB2 sequences. The new *Caloboletus* species is morphologically distinct and forms a well-supported, independent phylogenetic lineage, clearly separated from all previously described congeners. The Guizhou population of *Chenrenyua longispora* shows intraspecific morphological differences compared with the original protologue, and molecular data confirm its conspecificity. This study enriches the diversity of *Caloboletus* and extends the known geographical distribution and range of morphological variation of *C. longispora*. These findings further demonstrate that the species diversity of *Boletaceae* in southwestern China remains underestimated and highlight the importance of integrative taxonomic studies combining morphology and multilocus phylogenetic evidence.

## Introduction

Macrofungal diversity, complex morphology, and phylogenetic relationships have long received extensive attention from mycologists (Zeng et al. 2013; Wu et al. 2014, 2019; Xue et al. 2023; Dong et al. 2024, 2025; Qin et al. 2025; Wijesinghe et al. 2025; Wang et al. 2026). *Boletaceae* Chevall. is an important family of the order *Boletales*, and the vast majority of its species are ectomycorrhizal fungi with significant economic value and taxonomic research significance (Chen et al. 2016). *Caloboletus* Vizzini and *Chenrenyua* N.K. Zeng, T. Jiang, W.F. Lin & L.G. Hu are two well-defined genera within *Boletaceae*, both characterized by distinctive morphological features and supported as independent lineages by molecular phylogenetic studies. Members of *Caloboletus* are typically characterized by medium to large, fleshy basidiomata with a yellow hymenophore (tubes and pores) and olive-brown spore deposits; many species display a pronounced blue-staining reaction in the context when bruised or cut, and the flesh commonly has a distinctly bitter taste (Hellwig et al. 2002; Vizzini 2014; Zhao et al. 2014a; Zhang et al. 2017). *Caloboletus calopus* (Pers.) Vizzini was designated as the type species of the genus. *Chenrenyua* is a recently established genus of *Boletaceae*, characterized by boletoid basidiomata, a stipe covered with distinct warts, smooth basidiospores, and the absence of clamp connections. Phylogenetically, it forms an independent lineage within *Boletaceae*, distinct from other boletaceous genera such as *Caloboletus* and *Rubroboletus*. According to Index Fungorum (https://www.indexfungorum.org; accessed on 30 September 2026), *Caloboletus* includes 22 registered names, and *Chenrenyua* includes two registered names.

*Caloboletus* was established as a distinct genus from the broadly defined *Boletus* based on morphological characteristics and molecular phylogenetic evidence. Zhao et al. (2014a) conducted a phylogenetic analysis of *Caloboletus* and related taxa using four genetic markers, including the internal transcribed spacer (ITS) region, nuclear large subunit ribosomal RNA gene (nrLSU), translation elongation factor 1-alpha (*tef1*-α), and the largest subunit of RNA polymerase II (*rpb1*). Their results strongly supported *Caloboletus* as a distinct monophyletic lineage and recognized six species, including *C. calopus*, *C. radicans*, *C. firmus*, *C. inedulis*, *C. panniformis*, and *C. yunnanensis*. This study confirmed that *Caloboletus* corresponds to the former concept of *Boletus* sect. *Calopodes*. Members of this genus are mainly distributed in temperate and subtropical regions of the Northern Hemisphere and commonly form ectomycorrhizal associations with plants belonging to *Pinaceae* and *Fagaceae* (Zhao et al. 2014a). Subsequent studies using multilocus phylogenetic analyses further supported the independent phylogenetic status of *Caloboletus* and revealed additional species diversity, particularly in subtropical regions of China. These findings indicate that the taxonomy, evolutionary relationships, and biogeographic diversification of *Caloboletus* require further investigation with broader sampling and additional molecular data (Wu et al. 2016b; Zeng et al. 2019).

During ongoing surveys of macrofungi in Guizhou Province, southwestern China, several boletaceous specimens were collected. Morphological examination and phylogenetic analyses based on nrLSU, nrITS, *tef1*-α, and *rpb2* sequences revealed that one collection represents an undescribed species of *Caloboletus*, while another collection corresponds to *Chenrenyua longispora* with intraspecific morphological differences. Herein, the new *Caloboletus* species is formally described, supplementary notes on *C. longispora* are provided, their diagnostic morphological characters are illustrated, the fungal database is enriched, and the knowledge of the evolutionary relationships within these fungal genera is enhanced.

## Materials and methods

### Sample collection and morphological study

The specimens were obtained from Guizhou Province, southwestern China. Descriptions of macroscopic morphological features were derived from field observations and photographs taken *in situ*. Color descriptions followed the system proposed by Kornerup and Wanscher (1978). Following documentation of the macroscopic morphological features, each basidiome was bisected longitudinally to observe and record context discoloration resulting from injury. Basidiomata were subsequently dried in a forced-air oven at 45 °C for 10 h, placed in self-sealing plastic bags with silica gel desiccant, and temporarily deposited in a fungal resource preservation facility at 4 °C. For microscopic examination, dried specimens were sectioned and mounted in 5% KOH solution, followed by staining with 1% Congo red. Pileipellis sections were cut radially (perpendicular to the pileus surface) from the midpoint between the pileus center and margin. Stipitipellis and context sections were taken from the midpoint of the stipe (Li et al. 2011; Hosen et al. 2013; Zhu et al. 2015). Microscopic characters, including basidia, basidiospores, and cystidia, were examined and photographed under a Nikon Eclipse Ni compound microscope equipped with a Nikon Digital Sight 10 imaging system. For morphometric analyses, 30 basidiospores and 20 other structures were randomly selected for measurement. Basidiospore size is expressed as (*a*)*b*–*c*(*d*), where the range *b*–*c* represents the 95% measurement range, and “*a*” and “*d*” denote the extreme values. *Q* refers to the aspect ratio of a single basidiospore, while *Qm* refers to the mean *Q* value ± standard deviation. Voucher specimens examined in this study are deposited in the Herbarium of Cryptogams, Kunming Institute of Botany, Chinese Academy of Sciences (HKAS).

### DNA extraction, PCR amplification, and sequencing

Total genomic DNA was extracted from the specimens using the Ezup Column Fungal Genomic DNA Extraction Kit [Sangon Biotech (Shanghai) Co., Ltd., China]. For molecular identification and phylogenetic analysis, four molecular markers were selected, including nrLSU, nrITS, *tef1*-α, and *rpb2*. PCR amplification was performed using the following primer pairs: LR0R/LR5 (Vilgalys and Hester 1990) for nrLSU, ITS1/ITS4 (White et al. 1990) for nrITS, EF1-2F/EF1-2R (Rehner and Buckley 2005) for *tef1*-α, and bRPB2-6F/bRPB2-7.1R (Matheny 2005) for *rpb2*. PCR amplifications for the four molecular markers (nrLSU, nrITS, *tef1*-α, and *rpb2*) were carried out using 10 μL of 2× Taq PCR StarMix, 1 μL of each primer, 1 μL of DNA, and 7 μL of ddH_2_O, for a total volume of 20 μL. For nrLSU amplified with LR0R/LR5, the thermal profile consisted of an initial denaturation at 94 °C for 4 min, followed by 35 cycles of 94 °C for 40 s, 50 °C for 40 s, and 72 °C for 150 s, and a final extension at 72 °C for 10 min. For nrITS (ITS1/ITS4), the program comprised an initial denaturation at 95 °C for 5 min; 35 cycles of 94 °C for 45 s, 55 °C for 45 s, and 72 °C for 45 s; and a final extension at 72 °C for 8 min. Amplification of *tef1*-α with EF1-2F/EF1-2R used an initial denaturation at 94 °C for 4 min, followed by 35 cycles of 94 °C for 40 s, 50 °C for 40 s, and 72 °C for 60 s, and a final extension at 72 °C for 10 min. For *rpb2* using bRPB2-6F/bRPB2-7.1R, the conditions were as follows: initial denaturation at 95 °C for 5 min; 35 cycles of 95 °C for 30 s, 58 °C for 60 s, and 72 °C for 60 s; and a final extension at 72 °C for 7 min. The PCR products were detected using a 1% (w/v) agarose gel, purified according to the instructions provided with the Agarose Gel DNA Recovery Kit (CWBIO), and sequenced by Sangon Biotech (Shanghai) Co., Ltd.

### Sequence alignment and phylogenetic analyses

The nucleotide sequences generated in this study, together with additional homologous sequences retrieved from GenBank, were aligned using the online version of MAFFT 7.0 (http://mafft.cbrc.jp/alignment/server/; Katoh and Standley 2013). Alignments were manually checked and refined where necessary using BioEdit v. 7.0.9 (Hall 1999). A concatenated dataset of the four genes was constructed using SequenceMatrix v. 1.7.8 (Vaidya et al. 2011). A maximum likelihood (ML) phylogenetic analysis was conducted using RAxML-HPC2 v. 8.2.12 on the CIPRES Science Gateway platform (https://www.phylo.org/; Miller et al. 2010), with the GTR + CAT nucleotide substitution model implemented. Branch support was assessed using 1,000 bootstrap replicates (Stamatakis 2014). A Bayesian inference (BI) phylogenetic analysis was conducted using MrBayes v. 3.2.7a (Ronquist et al. 2012) on the CIPRES Science Gateway platform (https://www.phylo.org/; Miller et al. 2010). The best-fit nucleotide substitution model for each gene partition was selected using MrModeltest v. 2.0.1 based on the Akaike information criterion (AIC), with the GTR + I + G model ultimately selected for all gene regions. Three Markov chains were run, each undergoing 5 million generations of evolution. Sampling occurred every 100 generations until the mean standard deviation of split frequencies reached 0.01. After discarding the first 20% of the sampled trees, the remaining trees were aggregated to calculate posterior probabilities (PP) for each branch. The final phylogenetic tree was visualized and optimized using FigTree v. 1.4.0 software (Rambaut et al. 2014).

## Results

### Molecular data

**Figure 1.**
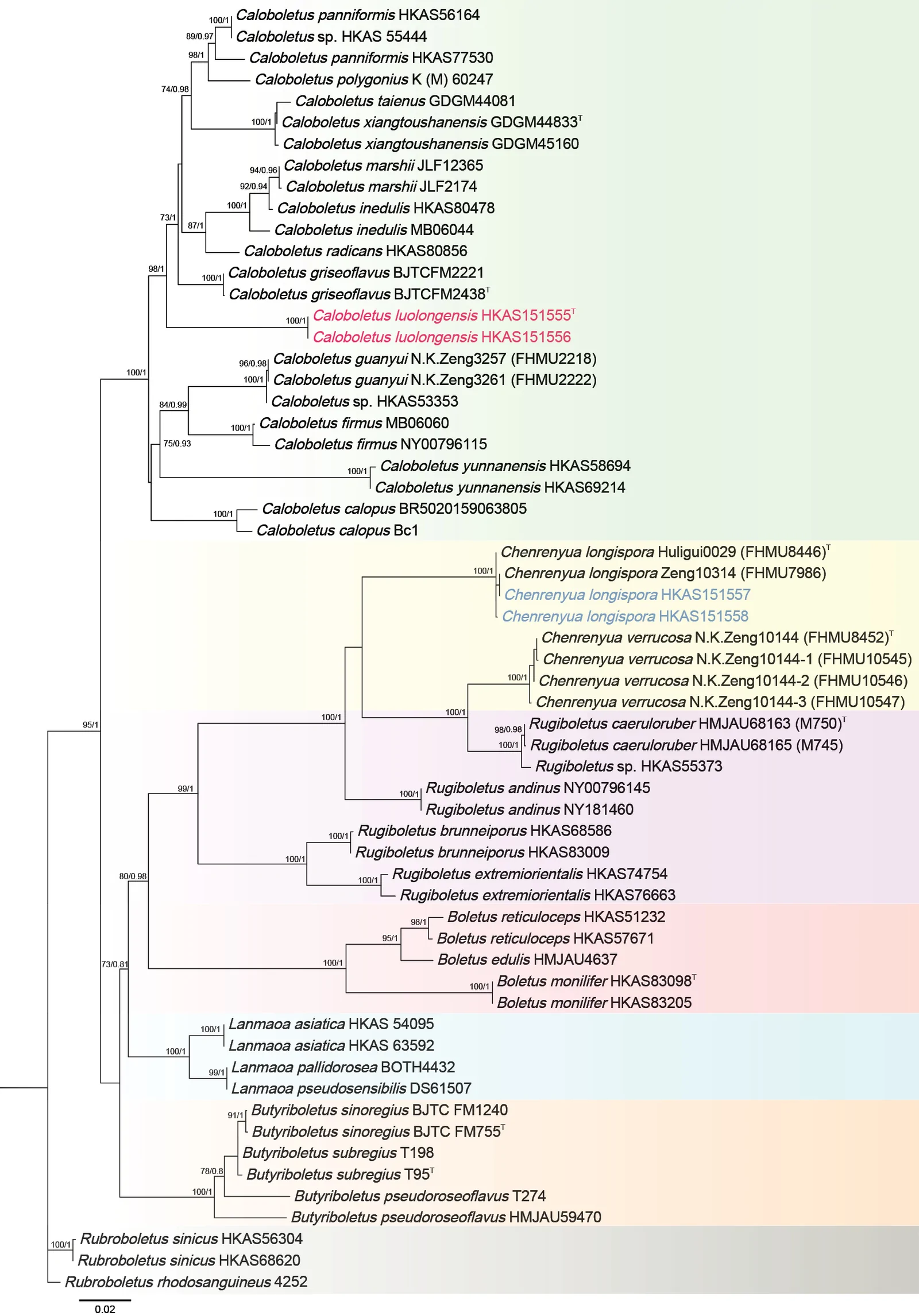
Phylogenetic tree generated using maximum likelihood and Bayesian inference methods based on an integrated dataset (nrLSU, nrITS, *tef1*-α, and *rpb2*). The figure depicts the ML tree. Bootstrap support values (BS ≥ 70%) and posterior probabilities (PP ≥ 0.90) are shown above the supported branches. New species are shown in red font to highlight their phylogenetic positions in the tree.

Phylogenetic analyses were performed using a combined dataset consisting of nrLSU, nrITS, *tef1*-α, and *rpb2* sequences. A total of 60 sequences were aligned, resulting in a dataset of 2,791 nucleotides (Table 1), corresponding to the following loci: 1–845 (nrLSU), 846–1500 (nrITS), 1501–2081 (*tef1*-α), and 2082–2791 (*rpb2*). The alignment contained 60 sequences with 2,791 columns, 1,113 distinct patterns, 843 parsimony-informative sites, 85 singleton sites, and 1,863 constant sites. Four sequences were newly generated in this study and deposited in GenBank. *Rubroboletus sinicus* (W.F. Chiu) Kuan Zhao & Zhu L. Yang and *R. rhodosanguineus* (Both) Kuan Zhao & Zhu L. Yang were used as outgroups. The phylogenetic trees constructed via ML and BI exhibited only minor discrepancies in statistical support values, with an overall high degree of topological congruence. Therefore, this study presents only the phylogenetic tree inferred through ML analysis (Fig. 1). Randomized Accelerated Maximum Likelihood (RAxML) analysis conducted on the merged dataset yielded an optimal tree with a final maximum likelihood value of −13254.167. Estimated nucleotide frequencies were A = 0.250, C = 0.250, G = 0.250, and T = 0.250. Estimated substitution rates were AC = 1.00000, AG = 4.44247, AT = 1.00000, CG = 1.00000, CT = 9.49793, and GT = 1.00000; the proportion of invariable sites was 0.440; and the gamma distribution shape parameter was α = 0.709.

**Table 1.** Names, vouchers, locations, and corresponding GenBank accession numbers of DNA sequences used in this study.

**Species**	**Voucher**	**Origin**	**GenBank accession numbers**				**Reference(s)**
			**nrLSU**	**nrITS**	***tef1*-α**	***rpb2***	
*Caloboletus calopus*	Bc1	Germany	AF456833	DQ679806	JQ327019	–	Zhang et al. (2017)
*Caloboletus calopus*	BR5020159063805	Belgium	KJ184554	KJ605655	KJ184566	–	Zhao et al. (2014a)
*Caloboletus firmus*	NY00796115	Belize	KJ605678	KJ605656	KJ619464	–	Zhao et al. (2014a)
*Caloboletus firmus*	MB06060	USA	KF030278	–	KF030408	–	Nuhn et al. (2013)
*Caloboletus griseoflavus*	BJTCFM2438*	China	OR655183	OR655147	OR659984	OR659935	Mao et al. (2023)
*Caloboletus griseoflavus*	BJTCFM2221	China	OR655182	OR655146	OR659983	OR659934	Mao et al. (2023)
*Caloboletus guanyui*	N.K.Zeng3257(FHMU2218)	China	MH879705	–	MH879732	MH879748	Chai et al. (2019)
*Caloboletus guanyui*	N.K.Zeng3261(FHMU2222)	China	MH879706	–	MH879733	MH879749	Chai et al. (2019)
*Caloboletus inedulis*	MB06044	USA	JQ327013	–	JQ327020	–	Halling et al. (2012)
*Caloboletus inedulis*	HKAS80478	USA	KJ605671	KJ605657	KJ619465	–	Zhao et al. (2014a)
***Caloboletus luolongensis***	HKAS151555*	**China**	**PX853910**	**PX854911**	**PX880080**	**PX880084**	**this study**
***Caloboletus luolongensis***	**HKAS151556**	**China**	**PX853911**	**PX854912**	**PX880081**	**PX880085**	**this study**
*Caloboletus marshii*	JLF2174	USA	KC812291	KC812290	–	–	Nuhn et al. (2013)
*Caloboletus marshii*	JLF12365	USA	PV335253	PP325812	–	–	Unpublished
*Caloboletus panniformis*	HKAS77530	China	KJ605670	KJ605661	KJ619469	–	Zhao et al. (2014a)
*Caloboletus panniformis*	HKAS56164	China	KJ605674	KJ605667	KJ619466	–	Zhao et al. (2014a)
*Caloboletus polygonius*	K(M)60247	Greece	KU317763	KU317753	–	–	Chai et al. (2019)
*Caloboletus radicans*	HKAS80856	France	KJ184557	KJ605662	KJ184569	–	Zhao et al. (2014a)
*Caloboletus* sp.	HKAS53353	China	KF112410	–	KF112188	KF112668	Wu et al. (2014)
*Caloboletus* sp.	HKAS 55444	China	KF112334	–	KF112165	KF112666	Wu et al. (2014)
*Caloboletus taienus*	GDGM44081	China	–	KY800420	–	–	Zhang et al. (2017)
*Caloboletus xiangtoushanensis*	GDGM44833*	China	KY800415	KY800421	KY800418	–	Zhang et al. (2017)
*Caloboletus xiangtoushanensis*	GDGM45160	China	KY800417	KY800423	KY800419	–	Zhang et al. (2017)
*Caloboletus yunnanensis*	HKAS69214*	China	KJ184556	KJ605663	KJ184568	–	Zhao et al. (2014a)
*Caloboletus yunnanensis*	HKAS58694	China	KJ605672	KJ605664	KJ619470	–	Zhao et al. (2014a)
*Chenrenyua longispora*	Huligui0029	China	PV938583	–	PV945447	–	Qin et al. (2025)
	(FHMU8446) *						
*Chenrenyua longispora*	Zeng10314	China	PV938584	–	PV945448	–	Qin et al. (2025)
	(FHMU7986)						
***Chenrenyua longispora***	**HKAS151557**	**China**	**PX853908**	**PX854909**	**PX880078**	**PX880082**	**this study**
***Chenrenyua longispora***	**HKAS151558**	**China**	**PX853909**	**PX854910**	**PX880079**	**PX880083**	**this study**
*Chenrenyua verrucosa*	N.K. Zeng10144	China	PV938581	–	PV945444	PV945459	Qin et al. (2025)
	(FHMU8452) *						
*Chenrenyua verrucosa*	N.K. Zeng10144-1	China	–	–	–	PV945460	Qin et al. (2025)
	(FHMU10545)						
*Chenrenyua verrucosa*	N.K. Zeng10144-2	China	–	–	PV945445	PV945461	Qin et al. (2025)
	(FHMU10546)						
*Chenrenyua verrucosa*	N.K. Zeng10144-3	China	PV938582	–	PV945446	PV945462	Qin et al. (2025)
	(FHMU10547)						
*Rubroboletus rhodosanguineus*	4252	USA	KF030252	–	KF030412	–	Nuhn et al. (2013)
*Rubroboletus sinicus*	HKAS68620	China	KF112319	KJ951991	KF112146	KF112661	Wu et al. (2014)
*Rubroboletus sinicus*	HKAS56304	China	KJ605673	KJ605666	KJ619483	–	Zhao et al. (2014b)
*Rugiboletus brunneiporus*	HKAS68586	China	KF112402	–	KF112197	–	Wu et al. (2016a)
*Rugiboletus brunneiporus*	HKAS83009	China	KM605133	–	KM605146	–	Wu et al. (2016a)
*Rugiboletus extremiorientalis*	HKAS76663	China	KM605135	–	KM605147	KM605170	Wu et al. (2016a)
*Rugiboletus extremiorientalis*	HKAS74754	China	KT990639	–	KT990832	KT990469	Wu et al. (2016b)
*Rugiboletus andinus*	NY181460	USA	MK601757	–	MK721111	MK766316	Kuo and Ortiz-Santana (2020)
*Rugiboletus andinus*	NY00796145	USA	MK601758	–	MK721112	MK766317	Kuo and Ortiz-Santana (2020)
*Rugiboletus* sp.	HKAS55373	China	KF112362	–	KF112303	KF112804	Wu et al. (2014)
*Rugiboletus caeruloruber*	HMJAU68163(M750)*	China	–	OR558331	OR566421	OR566426	Wang et al. (2024)
*Rugiboletus caeruloruber*	HMJAU68165(M745)	China	OR554014	OR558330	OR566420	OR566425	Wang et al. (2024)
*Boletus edulis*	HMJAU4637	Russia	KF112455	–	KF112202	KF112704	Wu et al. (2014)
*Boletus monilifer*	HKAS83098*	China	KM820807	–	–	–	Cui et al. (2016)
*Boletus monilifer*	HKAS83205	China	KM820806	–	–	–	Cui et al. (2016)
*Boletus reticuloceps*	HKAS51232	China	KT990537	–	KT990739	KT990376	Wu et al. (2016b)
*Boletus reticuloceps*	HKAS57671	China	KF112454	–	KF112201	KF112703	Wu et al. (2014)
*Lanmaoa asiatica*	HKAS 63592	China	KM605142	–	KM605152	KM605175	Wu et al. (2016a)
*Lanmaoa asiatica*	HKAS 54095	China	KM605141	–	KM605151	KM605174	Wu et al. (2014)
*Lanmaoa pseudosensibilis*	DS61507	USA	KF030257	–	KF030407	–	Nuhn et al. (2013)
*Lanmaoa pallidorosea*	BOTH4432	Thailand	–	–	MG897427	MG897437	Phookamsak et al. (2019)
*Butyriboletus sinoregius*	BJTC FM755*	China	OL721748	NR_185652	OL799252	OL771224	Fu et al. (2022)
*Butyriboletus sinoregius*	BJTC FM1240	China	OL721752	OK486135	OL799254	OL771226	Fu et al. (2022)
*Butyriboletus subregius*	T95*	China	–	–	OM285111	OM285109	Wang et al. (2022)
*Butyriboletus subregius*	T198	China	–	–	OM285112	OM285110	Wang et al. (2022)
*Butyriboletus pseudoroseoflavus*	T274	China	–	–	OL739124	OL739126	Wang et al. (2022)
*Butyriboletus pseudoroseoflavus*	HMJAU59470	China	–	OL604164	–	–	Wang et al. (2022)

Note: ‘–’ refers to missing data; ‘*’ refers to the type species.

Phylogenetic analyses based on the combined dataset recovered *Caloboletus* as a well-supported lineage. Within *Caloboletus*, two collections of the new species *C. luolongensis* (highlighted in red, HKAS151555 and HKAS151556) formed a strongly supported monophyletic lineage (BS = 100, PP = 1.00). It is sister to the clade comprising *C. griseoflavus* L. Fan, N. Mao & T.Y. Zhao 2023, and this combined clade is further nested within a larger group that includes *C. radicans* (Pers.) Vizzini 2014, *C. inedulis* (Murrill) Vizzini 2014, and *C. marshii* D. Arora, C.F. Schwarz & J.L. Frank 2014. This phylogenetic placement clearly separates *C. luolongensis* from all other known species of *Caloboletus*. The two newly sequenced specimens of *Chenrenyua longispora* (highlighted in blue, HKAS151557 and HKAS151558) form a fully supported monophyletic lineage (BS = 100, PP = 1.00), clustering together with the type specimen and other authenticated accessions of *C. longispora* N.K. Zeng, T. Jiang, W.F. Lin & L.G. Hu 2025. This *C. longispora* clade is sister to the clade of *C. verrucosa* W.F. Lin & L.G. Hu 2025, and these two *Chenrenyua* species together constitute a well-supported subclade, which is phylogenetically sister to the genus *Rugiboletus*.

BLAST searches against NCBI GenBank were carried out for the nrLSU, nrITS, *tef1*-α, and *rpb2* sequences of *Caloboletus luolongensis*. The five retrieved parameters (nucleotide identity, query coverage, E-value, GenBank accession number, and matched species) are summarized as follows: nrLSU: 97.88% identity, 100% query coverage, E-value = 0.0, MH213125, *Caloboletus* sp.; nrITS: 92.44% identity, 94% query coverage, E-value = 0.0, PZ517347, *Caloboletus* sp.; *tef1*-α: 95.30% identity, 97% query coverage, E-value = 0.0, MH614711, *Caloboletus* sp.; rpb2: 95.32% identity, 95% query coverage, E-value = 0.0, MG212626, *Caloboletus radicans*. The BLAST results indicated low sequence similarity with known species, supporting the status of the two taxa as independent new species.

### Taxonomy

#### Caloboletus luolongensis

Taxon classificationFungiBoletalesBoletaceae

Xing Mo, W.P. Zhang & X.H. Xu sp. nov.

C73E6809-763A-552A-A7F0-5D642C060410

862085

Fig. 2

##### Etymology.

Named after the collection site, Luolong Town, Yingzui County, Guizhou Province.

**Figure 2.**
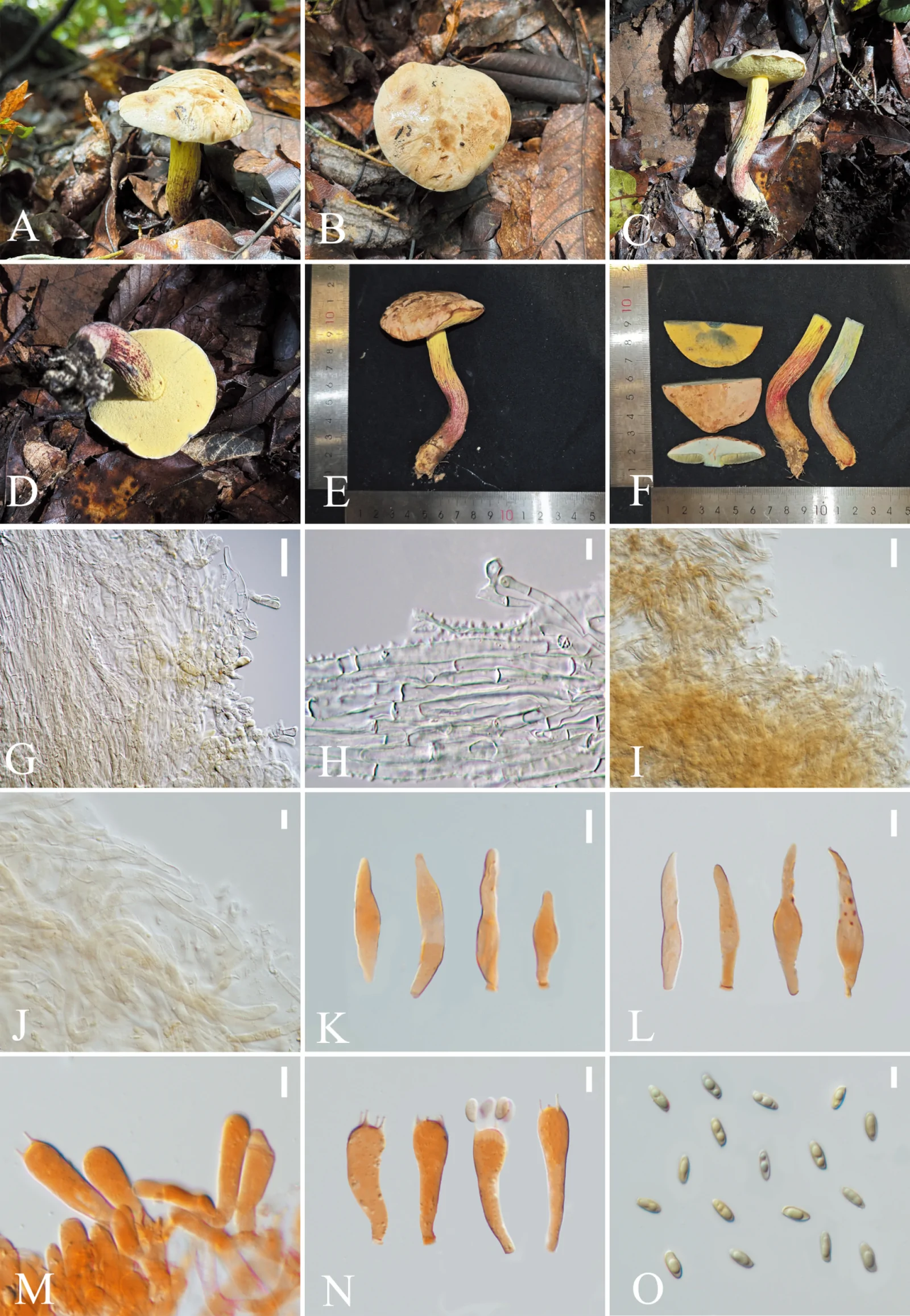
*Caloboletus luolongensis*. **A–F**. Basidiomata; **G**. Stipitipellis; **H**. Stipe trama; **I, J**. Pileipellis; **K**. Cheilocystidia; **L**. Pleurocystidia; **M, N**. Basidia; **O**. Basidiospores. All micrographs were taken from the holotype. Scale bars: 50 µm (**G, I**); 10 µm (**H, J–O**).

##### Holotype.

China • Guizhou Province: Zunyi City, Daozhen Gelaozu Miaozu Autonomous County, Luolong Town, Yingzui County; 29°6'40"N, 107°45'44"E, elev. 1,347 m, 24 September 2025, X. Mo and Z.Q. Qi, M83 (HKAS151555).

##### Description.

***Basidiomata*** medium-sized. Pileus 6–8 cm in diameter, convex to applanate, margin enrolled slightly; surface dry, pale yellow (1B4-6), at first rugulose especially near the margin, then subrugulose with age, finely tomentose to tomentose, unchanging in color when bruised. ***Context*** 1.3–1.7 cm thick, pale yellow (1B2-3), the lower part turning light blue quickly when injured. ***Hymenophore*** poroid, adnate or slightly depressed around apex of stipe. ***Pores*** angular, 1–2 mm in diameter, densely arranged, yellowish-white (30A2) to yellowish-brown (4A4), changing blue quickly when injured; tubes 4–6 mm in length, turning blue quickly when bruised. ***Stipe*** 8–9 × 1–2 cm, subcylindric, solid; surface dry, apical part bright yellow (2A5–2B5), transitioning to rose-red (12C5-6) towards the middle, and yellow at the base, covered with concolorous, longitudinal reticulation, changing bluish when injured; context yellowish to yellow, changing blue quickly when injured; basal mycelium white. Odor not distinctive. Taste not recorded.

***Basidiospores*** (11.04–)11.9–14.91(–15.77) × (4.41–)5.03–6.52(–7.14) μm [*Q* = (1.88–)2.06–3.07(–3.25) μm, *Qm* = 2.45 ± 0.30 μm], narrowly ellipsoid to subcylindrical with a slight suprahilar depression, colorless to pale yellow. ***Basidia*** 42–54 × 10–15 μm, clavate, thin-walled, four-spored, sterigmata 2–4 μm in length, colorless to yellowish in KOH. Hymenophoral trama boletoid. ***Cheilocystidia*** 37–53 × 6–10 μm, lageniform, thin-walled, colorless to pale yellow in KOH. ***Pleurocystidia*** 55–85 × 7–12 μm, fusiform to lanceolate, thin-walled, colorless to yellowish in KOH. ***Pileipellis*** composed of interwoven hyphae, 4–8 μm wide, and consistently encrusted with brown extracellular pigment. ***Stipitipellis*** consists of pale yellow, parallel hyphae, 7–15 μm wide. All hyphae show no reaction in Melzer’s reagent. Clamp connections absent in all tissues.

##### Habitat.

Solitary or scattered in mixed forests of *Fagaceae* (*Quercus* spp., *Castanopsis* spp.) and *Pinaceae* (*P. massoniana*).

##### Distribution.

China, Guizhou Province.

##### Additional material examined.

China • Guizhou Province: Zunyi City, Daozhen Gelaozu Miaozu Autonomous County, Luolong Town, Yingzui County; 29°6'40"N, 107°45'44"E, elev. 1347 m, 24 September 2025, X. Mo and Z.Q. Qi, M77 (HKAS151556).

##### Notes.

*Caloboletus luolongensis* is characterized by a rugose pileus with a slightly incurved margin, a longitudinally reticulate stipe that is rose-red over its middle and lower parts, and a context that bruises blue when injured. Morphologically, *C. luolongensis* is highly similar to *C. panniformis* (Taneyama & Har. Takah.) Vizzini and *C. polygonius* (A.E. Hills & Vassiliades) Vizzini. However, *C. panniformis* exhibits a reticulate surface on the upper half that becomes pruinose downward, with a reddish-brown (9D5) tint toward the base, and the reticulation and pruina appear dull (Takahashi et al. 2013; Chai et al. 2019); *C. polygonius* is characterized by a dry pileus that cracks into polygonal scales at maturity and a yellow stipe covered with dense reddish fibrils but lacking reticulation (only a faint reticulum occasionally at the stipe apex) (Hills and Vassiliades 1999). Among other species of *Caloboletus*, *C. taienus* and *C. xiangtoushanensis* exhibit pores ranging from deep red and brownish-red to ruby (10C8–12C8) and deep orange to reddish-orange, respectively (Zhang et al. 2017). The cap of *Caloboletus griseoflavus* is grayish-white to grayish-brown, and the stem is yellow (Mao et al. 2023). *C. radicans* has large basidiomata (pileus 8–30 cm in diameter) and a root-like base extending into the soil (Bon et al. 1987). The base of the stipe of *Caloboletus marshii* is often bulbous, and its surface is completely devoid of reticulation. *C. inedulis* has conspicuous red reticulation on the surface of the stipe and a cap that commonly cracks with age (Zhao et al. 2014a).

#### Chenrenyua longispora

Taxon classificationFungiBoletalesBoletaceae

N.K. Zeng, T. Jiang, W.F. Lin & L.G. Hu

5B7B251F-DB39-5F4D-A929-39502BA512D1

860310

Fig. 3

##### Description.

***Basidiomata*** of the Guizhou collections small to medium-sized. Pileus 3–5 cm in diameter, hemispherical to convex, incurved or suspended at the margin, surface dry, tomentose, usually wrinkled when old, light brown or brownish yellow (5C6–5D6). ***Context*** 0.8–1.3 cm thick in the center of the pileus, pale yellow (2A2) or light reddish brown (6C4), unchanging in color when injured. ***Hymenophore*** poroid, with a depression around the apex. ***Pores*** nearly round, minute, densely arranged, orange-red, deep orange-red to brick-red (8D5–8D8), unchanging in color when injured; tubes 0.3–1 cm in length, yellowish (2A4), unchanging in color when injured. ***Stipe*** 5–8 × 0.5–2.5 cm, cylindrical or obclavate, surface yellowish white (1A2) to greyish yellow (4B4), with concolorous or darker (orange-red) granulose squamules; context pale yellow to light yellow mixed with brown tinge, unchanging in color when injured. Basal mycelium white. Odor and taste not recorded.

**Figure 3.**
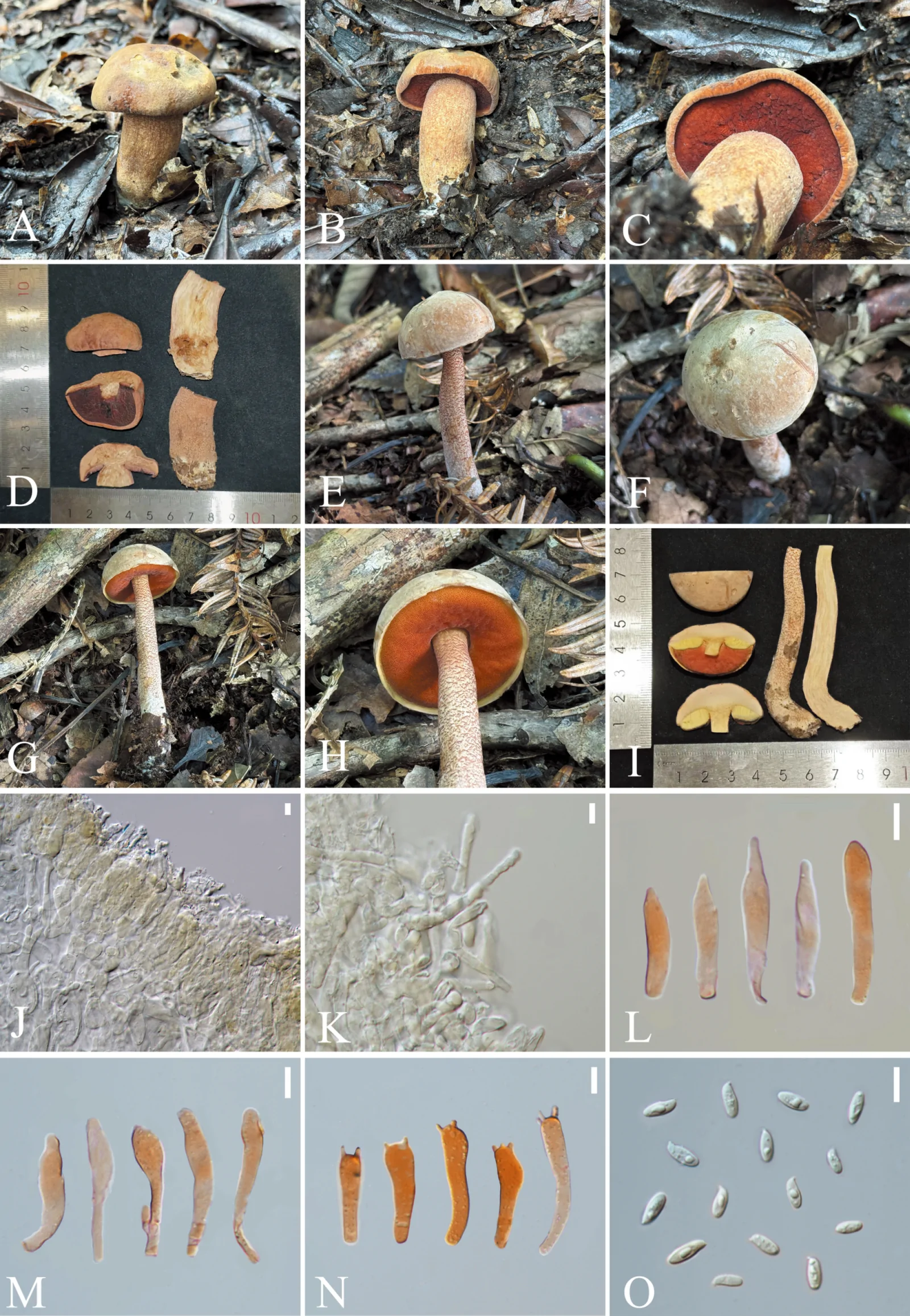
*Chenrenyua longispora*. **A–I**. Basidiomata (**A–D**. HKAS151557; **E–I**. HKAS151558); **J**. Stipitipellis; **K**. Pileipellis; **L**. Cheilocystidia; **M**. Pleurocystidia; **N**. Basidia; **O**. Basidiospores. Scale bars: 10 µm (**J–O**).

***Basidiospores*** (8.33–)8.80–14.00(–14.62) × (3.60–)3.80–5.50(–5.73) μm [*Q* = (1.66–)1.83–3.10(–3.27) μm, *Qm* = 2.83 ± 0.39 μm], elliptic-fusiform to subfusiform, with a weak or distinct suprahilar depression, smooth. ***Basidia*** 26–50 × 6–10 μm, clavate, thin-walled; four-spored, sterigmata 2–5 μm in length, colorless to yellowish in KOH. Hymenophoral trama boletoid, composed of hyaline, filamentous hyphae, 4–7 μm wide. ***Cheilocystidia*** 24–41 × 4–9 μm, fusiform or subfusiform, thin-walled, colorless in KOH. ***Pleurocystidia*** 33–49 × 5–9 μm, lageniform or clavate to fusiform, thin-walled, colorless to yellowish in KOH. ***Pileipellis*** an interwoven trichodermium, composed of yellowish-brown hyphae, usually filamentous sometimes slightly inflated, occasionally covered with granular crustation, 3–6 μm wide; terminal cells 34–50 × 3–9 μm, usually clavate or subclavate. Pileal trama made up of hyphae 4–8 μm in diameter, colorless to pale yellow in KOH. ***Stipitipellis*** hymeniform 80–100 μm thick, composed of thin-walled hyphae, yellowish in KOH, terminal cells 38–40 × 7–13 μm. Stipe trama composed of parallel hyphae 4–10 μm wide. Clamp connections absent in all tissues.

##### Distribution.

Zhejiang Province of China; Guizhou Province of China.

##### Specimens examined.

China • Guizhou Province: Zunyi City, Daozhen Gelaozu Miaozu Autonomous County; 29°6'35.60"N, 107°45'42.43"E, elev. 1,340.33 m, 24 September 2025, X. Mo and Z.Q. Qi, M85 (HKAS151557); • same locality and date, M92 (HKAS151558).

##### Notes.

The collections from Daozhen, Guizhou, were initially considered to represent an undescribed species of *Rugiboletus*. However, multilocus phylogenetic analyses based on nrITS, nrLSU, *tef1*-α, and *rpb2* demonstrated that the two specimens (HKAS151557, HKAS151558) were firmly nested within the monophyletic clade of *Chenrenyua longispora*, together with its holotype and published reference sequences, with maximum nodal support (BS = 100, PP = 1.00). *Chenrenyua longispora* HKAS151557 features a wrinkled cap surface with orange-red pores and a thick, cylindrical stipe bearing squamules concolorous with the stipe, whereas *Chenrenyua longispora* HKAS151558 exhibits a cap surface covered with a white powdery substance, orange-red pores, and a slender, obclavate stipe with squamules matching the color of the pores. In contrast, the hymenophore and context of *Chenrenyua longispora* FHMU8446 (holotype) quickly turn blue when injured, and it has slender, fusiform basidiospores. Subtle morphological differences were observed between the examined materials and the original protologue. Additional morphological observations and newly generated nrITS and *rpb2* sequences are provided for this species.

## Discussion

The discovery of *Cal. luolongensis* and newly documented supplementary specimens of *Chenrenyua longispora* enrich the known species diversity of boletes in southwestern China. Morphological evidence, supported by substantial genetic distances in multilocus (nrLSU, nrITS, *tef1*-α, and *rpb2*) phylogenetic analyses, clearly distinguishes *Cal. luolongensis* from all previously described congeners within *Caloboletus*, while the Daozhen collections of *Ch. longispora* show subtle morphological variations compared with its original protologue. *Caloboletus luolongensis* is nested within the core clade of *Caloboletus* and exhibits a distinct genetic distance in the phylogenetic tree (Fig. 1). This result is consistent with the well-supported monophyly of *Caloboletus* under the multilocus phylogenetic framework (Vizzini 2014; Wu et al. 2014), further validating the appropriate generic placement of this new species. Specimens of *Chenrenyua longispora* from Daozhen cluster firmly within the monophyletic clade of this species, closely related to *Ch. verrucosa*. The newly generated nrITS and *rpb2* sequences and morphological observations supplement the known variation of *Ch. longispora*. *Chenrenyua* is phylogenetically sister to *Rugiboletus*.

The region where *Cal. luolongensis* and the *Ch. longispora* materials were collected is located in the mountainous areas of southwestern China, recognized as one of the key hotspots of ectomycorrhizal fungal diversity in the Northern Hemisphere (Tedersoo et al. 2012). Consequently, the documentation of *Cal. luolongensis* and supplementary data for *Ch. longispora* not only enrich the species composition of their respective genera but also provide new evidence for understanding the evolutionary and diversification patterns of *Boletaceae* in East Asia.

## Supplementary Material

XML Treatment for Caloboletus luolongensis

XML Treatment for Chenrenyua longispora

## Additional information

### Artificial Intelligence (AI) use

The authors accept full responsibility for the content of the manuscript, including the disclosure of any use of AI.

No AI tools were used in the preparation of this manuscript.

### Author ORCIDs

X. Mo https://orcid.org/0009-0003-0295-5062

Z.-Q. Qi https://orcid.org/0009-0000-9576-0237

Z.-Q. Zhang https://orcid.org/0009-0003-1195-3595

R. Dai https://orcid.org/0009-0008-0670-7759

Y.-D. Li https://orcid.org/0009-0005-0470-0850

Y. Pei https://orcid.org/0000-0001-7730-4561

J.-W. Li https://orcid.org/0009-0002-4342-5837

T.-C. Wen https://orcid.org/0000-0003-1744-5869

W.-P. Zhang https://orcid.org/0000-0002-2029-6844

N.-K. Zeng https://orcid.org/0000-0001-7651-4524

X.-H. Xu https://orcid.org/0000-0002-6602-4246

### Data availability

All of the data that support the findings of this study are available in the main text or Supplementary Information.

## Supplementary materials

Supplementary material 1 ABI original files Data type zip https://binary.pensoft.net/file/1820924This dataset is made available under the Open Database License (http://opendatacommons.org/licenses/odbl/1.0/). The Open Database License (ODbL) is a license agreement intended to allow users to freely share, modify, and use this Dataset while maintaining this same freedom for others, provided that the original source and author(s) are credited. Xing Mo, Zhuo-Qiong Qi, Zhi-Qi Zhang, Rong Dai, Ya-Dong Li, Yun Pei, Jing-Wei Li, Ting-Chi Wen, Wan-Ping Zhang, Nian-Kai Zeng, Xiu-Hong Xu
